# Roots of the Resurrection Plant *Tripogon loliiformis* Survive Desiccation Without the Activation of Autophagy Pathways by Maintaining Energy Reserves

**DOI:** 10.3389/fpls.2019.00459

**Published:** 2019-04-25

**Authors:** Pauline Asami, Thusitha Rupasinghe, Lalehvash Moghaddam, Isaac Njaci, Ute Roessner, Sagadevan Mundree, Brett Williams

**Affiliations:** ^1^Centre for Tropical Crops and Biocommodities, Queensland University of Technology, Brisbane, QLD, Australia; ^2^Metabolomics Australia, School of BioSciences, The University of Melbourne, Melbourne, VIC, Australia; ^3^Biosciences Eastern and Central Africa-International Livestock Research Institute, Nairobi, Kenya

**Keywords:** energy metabolism, sucrose, T6P, tolerance, *T. loliiformis*, source/sink

## Abstract

Being sessile, plants must regulate energy balance, potentially via source-sink relations, to compromise growth with survival in stressful conditions. Crops are sensitive, possibly because they allocate their energy resources toward growth and yield rather than stress tolerance. In contrast, resurrection plants tightly regulate sugar metabolism and use a series of physiological adaptations to suppress cell death in their vegetative tissue to regain full metabolic capacity from a desiccated state within 72 h of watering. Previously, we showed that shoots of the resurrection plant *Tripogon loliiformis*, initiate autophagy upon dehydration as one strategy to reinstate homeostasis and suppress cell death. Here, we describe the relationship between energy status, sugar metabolism, trehalose-mediated activation of autophagy pathways and investigate whether shoots and roots utilize similar desiccation tolerance strategies. We show that despite containing high levels of trehalose, dehydrated Tripogon roots do not display elevated activation of autophagy pathways. Using targeted and non-targeted metabolomics, transmission electron microscopy (TEM) and transcriptomics we show that *T. loliiformis* engages a strategy similar to the long-term drought responses of sensitive plants and continues to use the roots as a sink even during sustained stress. Dehydrating *T. loliiformis* roots contained more sucrose and trehalose-6-phosphate compared to shoots at an equivalent water content. The increased resources in the roots provides sufficient energy to cope with stress and thus autophagy is not required. These results were confirmed by the absence of autophagosomes in roots by TEM. Upregulation of *sweet* genes in both shoots and roots show transcriptional regulation of sucrose translocation from leaves to roots and within roots during dehydration. Differences in the cell’s metabolic status caused starkly different cell death responses between shoots and roots. These findings show how shoots and roots utilize different stress response strategies and may provide candidate targets that can be used as tools for the improvement of stress tolerance in crops.

## Introduction

Drought is a major abiotic stress that significantly reduces the productivity of crops. Coupled with increased temperatures, drought can reduce crop yield by up to 50% (Lamaoui et al., 2018). Forecasts suggest that only the most resilient species will continue to yield in future climatic conditions (Saab, 2016). A group of resilient plants termed desiccation tolerant plants or resurrection plants can withstand water loss to a desiccated state and regain full metabolic capacity within 72 h of rehydration. These plants are generally slow growing and are found on rocky outcrops or in nutrient poor soils that are subjected to wet and dry periods on a daily or even hourly basis. To survive, resurrection plants must compromise their growth and survival with water and energy resources. Unlike sensitive plants, resurrection plants tightly regulate their water loss by having reduced numbers of stomata and leaf surface area, the presence of bulliform cells that act as water reservoirs and the protection of proteins by accumulation of sugars (Gaff and Oliver, 2013; Kabaraschi et al., 2015). They are also agile in their responses to the environment, often rapidly responding to even the smallest amounts of water loss to implement the appropriate stress response. For example, resurrection plants can shutdown photosynthesis during the early stages of dehydration, they also accumulate and transport stress associated-metabolites when hydrated (Urano et al., 2017). Conversely, desiccation sensitive plants utilize water reserves and photosynthesise even into the dehydrated state (Mundree et al., 2002). Desiccation tolerance in vegetative tissue in angiosperms is rare, existing in approximately 135 species. Despite this exclusivity, at the genetic level all orthodox seed producing angiosperms tolerate desiccation in at least one stage of their life cycle, seed maturation (Farrant, 2000). For instance, *Arabidopsis thaliana* seeds are desiccation tolerant but lose their tolerance upon germination (Maia et al., 2011). Therefore, the differences observed between sensitive and tolerant species are likely associated with regulation of stress responses. To achieve desiccation tolerance, resurrection plants suppress growth and reproduction during dehydration (Scott and Sciences, 2000).

The resurrection grass *Tripogon loliiformis* is native to Australia and can withstand desiccation and returning to full metabolic capacity within 72 h of watering (Williams et al., 2015; Karbaschi et al., 2016). Amongst physiological and structural adaptations, recent studies show that *T. loliiformis* may tolerate desiccation by tightly regulating programmed cell death (PCD) pathways, partially through regulation of nitrogen metabolism, early shutdown of photosynthesis, suppression of senescence, efficient reactive oxygen species (ROS) scavenging systems as well as sugar metabolism (Williams et al., 2015; Asami et al., 2018). More recently, the tight regulation of cell death and autophagy pathways by sugar metabolism, including trehalose, has gained recognition as a putative tolerance mechanism (Griffiths et al., 2014; Williams et al., 2015; Zhu et al., 2015; Asami et al., 2018).

Trehalose is one of the most studied desiccation-associated stress effectors and is found in bacteria, yeast, fungi and several plants (Elbein et al., 2003). Apart from *T. loliiformis*, several resurrection plants accumulate low levels of trehalose during drying (Ghasempour et al., 1998; Yobi et al., 2017). Trehalose is thought to play a protective role in plants against various environmental stresses, however, the molecular mechanisms of this protection remain unknown (Elbein et al., 2003). *Selaginella lepidophylla* accumulates trehalose to levels equivalent to 12% of the plant’s dry weight during desiccation and this accumulation is associated with the protection of proteins and membrane structures (Goddijn and Van Dun, 1999). In yeast cells, high levels of trehalose are required for long term but not short term survival. *Saccharomyces cerevisiae* utilizes the metabolic effects of trehalose biosynthetic intermediates (Tapia et al., 2015) rather than trehalose as an energy source to enhance desiccation tolerance. Exogenous application of trehalose was shown to trigger autophagy in plants (Williams et al., 2015; Li et al., 2016).

Dehydration-induced, early shut down of photosynthesis by *T. loliiformis* causes an energy deficit (Williams et al., 2015). When starved, cells implement a range of responses to sustain themselves. A universal response is to shut down energy consuming processes and preserve energy through the accumulation of sugars. These sugars play the dual role of energy stores as well as cytoprotective agents that shield protecting macromolecules and membranes from damage as well as scavenge ROS. Resurrection plants accumulate distinct sugar profiles depending on the species. For example, dehydrating *Xerophyta viscosa* plants accumulate sucrose and raffinose family oligosaccharides (Peters et al., 2007) while *Craterostigma plantagineum* accumulates octulose (Norwood et al., 2000). *T. loliiformis* accumulates low levels of trehalose (Williams et al., 2015).

Sugar levels are directly related to the cell’s energy status and are used as substrates for intermediary metabolism and as signaling molecules to link carbon metabolism with plant growth/development. The key regulators of energy homeostasis are signaling pathways involving target of rapamycin (TOR) and energy sensor sucrose non-fermenting 1-related kinase 1 (snRK1) proteins (Yadav et al., 2014). SnRK1 activity is regulated via trehalose intermediate, trehalose-6-phosphate, which serves as an indicator of sucrose availability (Yadav et al., 2014). The detection of T6P by SnRK1 modulates source-sink interactions and can also regulate plant adaptation pathways. Absence of T6P indicates an energy deficit and triggers the activation of snRK1 and catabolic pathways such as autophagy (Nunes et al., 2013).

Sugar accumulation is important for resurrection plant survival. However, whether sugar accumulation is a direct strategy or consequence of other tolerance mechanisms and insights on how these mechanisms promote survival in *T. loliiformis* particularly the role of source-sink flux remains unknown. Transcriptome analysis of dehydrating and desiccated *T. loliiformis* plants showed that autophagy is induced in shoots but not roots. Here we show that the differential response of *T. loliiformis* shoots and roots to dehydration is mediated via energy reserves and their effects on metabolic signaling pathways. Dehydrated roots maintain high energy status by accumulating sucrose, T6P and T6P/SUC. These energy resources suppress snRK1 activation and autophagy. Shoots on the other hand sacrifice their energy reserves to maintain root vitality and translocate significant amounts of their sugars to the roots. The translocation of sugars from the shoots results in lower energy levels which triggers autophagy to maintain homeostasis and cell vitality. This is reminiscent of long-term drought responses engaged by sensitive plants. We postulate that the accumulation of stress-associated metabolites in the hydrated state enables resurrection plants to activate long-term rather than short-term stress pathways during the initial stages of dehydration. These results provide much needed insight into desiccation tolerance through source/sink dialogue and may highlight potential gene targets for the development of drought tolerant crops.

## Materials and Methods

### Plant Materials and Cultivation

*Tripogon loliiformis* plants were germinated from seeds collected from a single plant and grown in a chamber at 27°C and 16 h photoperiod. Twenty one, 65 mm pots having several plants were grown for 2 months and later watered to saturation. Hydrated controls were randomly collected in three replicates, 1 day post-watering. Water was withheld in the remaining plants until they were air dry and their relative water content (RWC) dropped below 10%. This was equivalent to dehydration. Triplicate samples were collected once the plants were at 80, 60, 40, and <10% RWC. Rehydration was done by watering desiccated plants and samples were collected after 48 h. The percentage RWC was determined on *T. loliiformis* shoots and roots and was calculated according to Barrs and Weatherley (1962) using the formula (RWC (%) = ((Fresh Weight—Dry Weight)/(Turgid Weight—Dry Weight)) x 100). All the shoot and root samples were snap frozen in liquid nitrogen and stored at -80°C until RNA was isolated.

#### Total RNA Extraction and High Throughput Sequencing

For sequencing analysis, total RNA was isolated from the shoot and root tissue of triplicate hydrated, dehydrating, dehydrated and rehydrated *T. loliiformis* plants using a modified Trizol (Invitrogen) and spin column (Qiagen) method. The Bioanalyzer (Agilent technologies) was used to verify RNA integrity and quality. For library preparation, polyadenlyated RNA was enriched, chemically fragmented and cDNA was synthesized using an Illumina RNA-seq kit according to manufacturer’s instructions. Sequencing of the cDNA libraries was performed at Texas A&M AgriLife Genomics and Bioinformatics service, United States using an Illumina HiSeq 2500 Sequencer (Illumina, Inc.). 100 bp single-read sequences were collected.

#### RNA-seq Analysis

Quality control on the sequences was performed. Primer and barcode sequences were removed by trimming. A *de novo* assembled and blast annotated, this *T. loliiformis* transcriptome assembly served as a reference for RNA-Seq profiling of the independent cDNA libraries (Williams et al., 2015). Over 80% of the reads from each sample were mapped. All data sets were paired and used in an *in silico* microarray experiment using CLC genomics workbench. Using the Hydrated sample as a reference, each data set was enriched for genes that had a fold change _ 2 or _ -2. Prior to analysis the data were subjected to quantile normalization. Essentially, the distributions of the expression values for each replicate were used to create a common target distribution. The targeted distribution was then used to calculate normalized expression values.

### Validation of atg Transcripts Using Quantitative Real-Time PCR (qRT-PCR)

Approximately 0.8 μg of total RNA was used to generate cDNA using 100 pmol oligo (dT) primer and superscript III reverse transcriptase (Invitrogen). SYBR Green PCR Master Mix Kit (Applied Biosystems) was used to perform quantitative PCR according to manufacturer’s instructions. Standard cycling parameters coupled with 300 mM primer, a dilution of 1/100 cDNA and ViiA7 Real-Time PCR system were used. ATG specific primers were designed using Primer3 bioinformatics software as shown in Supplementary Table S2. ExpressionSuite software (Life Technologies) was used to perform analysis of the data. Normalization was done using the *T. loliiformis* homolog of *Arabidopsis* Actin identified from the annotated transcriptome. Hydrated tissues were used as controls for fold change calculation.

### Measurement of Metabolites

#### GC–MS and LCMS Analysis of Metabolites in Hydrated, Dehydrating, and Desiccated *T. loliiformis* Shoots

To analyze changes in *T. loliiformis* metabolite accumulation during dehydration GCMS and LCMS was performed. Two month old hydrated, dehydrated (80, 60, and 40% RWC) and desiccated < 10% RWC plants were harvested, snap-frozen in liquid nitrogen and lyophilised overnight. Following lyphilozation, the dry weight was measured for normalization and the samples were ground to a powder using a Qiagen tissue lyser (2 min × 1 min). Metabolites were extracted using water and chloroform and analyzed by mass spectrophotometer. The mass spectrophotometry data was annotated using a pre-existing mass spectra repository and databases as described by Dias et al. (2016). Electron ionization of mass spectra were ecorded at a scanning range of 30–650 m/z. All experiments were conducted using three biological replicates.

### Transmission Electron Microscopy

#### Detection of Autophagosomes by TEM

To assess whether shoots and roots trigger autophagy pathways, hydrated and dehydrated mid sections of both leaf and root tissues were viewed under transmission electron microscopy (TEM) for the presence of autophagosomes. Hydrated leaf and root samples from 3 month old glasshouse grown *T. loliiformis* plants were harvested together with dehydrated samples. For TEM, both root and leaf sections were fixed in 3% glutaraldehyde in 0.1 M sodium cacodylate buffer followed by post-fixation in 1% osmium tetroxide in 0.1 M sodium cacodylate buffer. Samples were rinsed in UHQ water and dehydrated through a graded series of acetone washes and embedded in Embed-812 resin. Ultrathin sections were cut using a Leica UC7 ultramicrotome (Leica Microsystems, Wetzlar, Germany) and imaged with a JEOL JEM-1400 TEM at an accelerating voltage of 80 kV.

### Statistical Analysis

Data was collected and analyzed using Minitab software Version 17. ANOVA was carried out to determine the significance at *P* < 0.05.

## Results

### *T. loliiformis* Triggers Autophagy in Shoots but Not Roots During Drying

Previously, we showed that *T. loliiformis* uses trehalose metabolism to trigger autophagy in shoots during dehydration for the removal of damaged and unwanted proteins as well as to prevent cell death. To determine whether *T. loliiformis* roots use a similar strategy to survive desiccation we performed RNA-seq analysis of roots from hydrated, dehydrating and desiccated *T. loliiformis* plants (Williams et al., 2015). Prior to analyzing the root transcriptome, and to ensure that a fair comparison could be made, we assessed whether the roots and shoots contained an equivalent RWC at each dehydration point. Table 1 shows that the RWC of shoots and roots was similar across the dehydration points. To assess whether *T. loliiformis* roots regulate cell death pathways during dehydration at the transcriptional level in a similar manner to shoot we analyzed the transcript accumulation of a suite of cell death-associated genes (Williams et al., 2015; Costa et al., 2017). As shown in Table 2, dehydrating and desiccated *T. loliiformis* shoots and roots displayed similar regulation of apoptotic-like cell death as well as senescence-associated genes. Unlike the shoots which showed an increase during dehydration, autophagy-associated transcripts did not accumulate in dehydrating roots (Table 2 and Supplementary Table S1). Additionally, unfolded protein response (*upr*) genes were downregulated in shoots and upregulated in roots.

**Table 1:** Dehydrating *T. loliiformis* shoots and roots contain a similar relative water content.

	10 RWC	40 RWC	60 RWC	80 RWC
Shoots	7.62 ± 1.60	39.62 ± 1.67	61.83 ± 2.49	78.25 ± 2.56
Roots	10.03 ± 1.43	37.81 ± 0.59	61.75 ± 0.32	78.99 ± 2.58

**Table 2:** Fold change of cell death associated genes expressed at different desiccation states between shoots and roots of *Tripogon loliiformis* 60, 40, <10 RWC and rehydration.

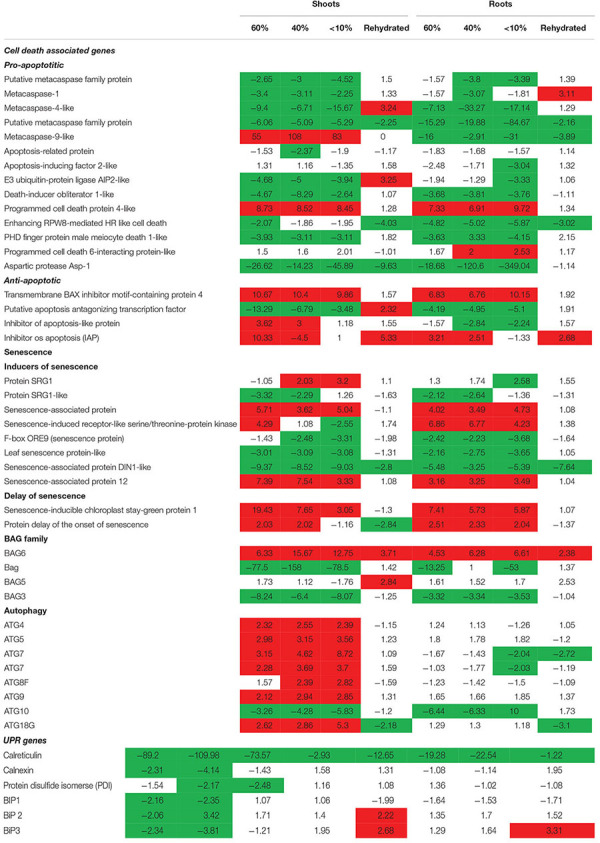

*P < 0.05. Cells denoted in red and green indicate increased and decreased transcript accumulation compared to hydrated controls*.

**Figure 1:**
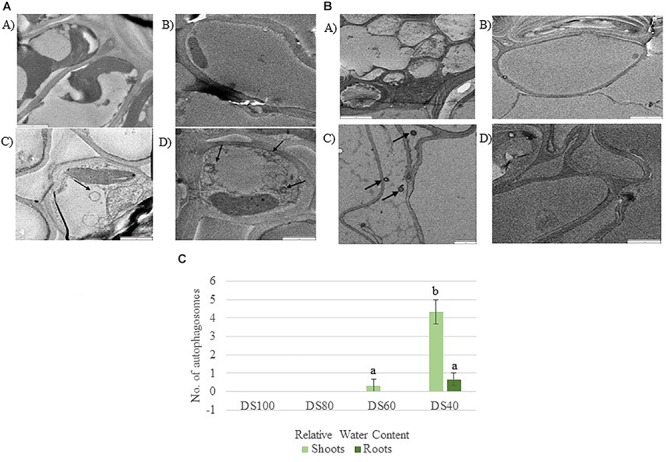
**(A)** Autophagosomes in *Tripogon loliiformis* shoots during dehydration. (A) Hydrated shoots (B) 80 RWC (C) 60 RWC (D) 40 RWC. **(B)** Few autophagosomes observed in *T. loliiformis* roots during dehydration. (A) Hydrated shoots (B) 80 RWC (C) 60 RWC (D) 40 RWC. **(C)** Comparison in autophagosome number between dehydrated *T. loliiformis* shoots and roots. *P* < 0.05. Samples denoted with the same letter were not statistically different from each other using a *P*-value < 0.05.

The transcriptome data suggest that autophagy pathways are not induced in dehydrated and desiccated *T. loliiformis* roots. To eliminate the possibility of post-transcriptional regulation of autophagy in roots and determine whether autophagy was occurring at the physiological level we performed TEM and looked for the presence of autophagosomes. Autophagosomes are double-membrane vesicles formed during autophagy that traffic cellular components to the plant vacuole for degradation and recycling. Hydrated, dehydrating (60 and 40% RWC) and desiccated shoots and roots were sectioned and viewed by electron microscopy. In contrast to shoot samples which contained numerous double-membraned vesicles, little to no autophagosomes were observed in roots at the same dehydration points (Figure 1A–C).

### Roots Maintain High Energy Reserves to Suppress Autophagy During Dehydration

**Figure 2:**
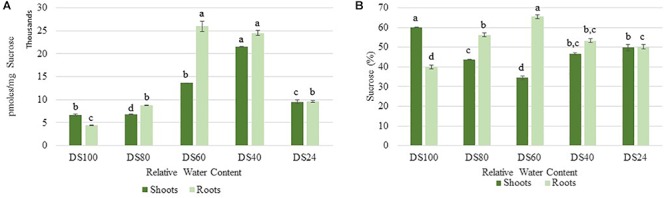
**(A)** Sucrose accumulation between shoots and roots of *T. loliiformis* during dehydration. *P* < 0.05. **(B)** Percentage sucrose accumulation between shoots and roots of *T. loliiformis* during dehydration. Samples denoted with the same letter were not statistically different from each other using a *P*-value < 0.05.

Cells sense energy and sucrose levels via the trehalose intermediate Trehalose-6-phosphate (T6P). Accordingly, T6P is positively correlated to sucrose availability and both sugars work together to maintain cellular homeostasis during stress (Yadav et al., 2014). High levels of T6P indicate an energy surplus to promote anabolic and inhibit catabolic pathways via suppression of the sucrose non-fermenting-related protein kinase 1 (SnRK1). To determine whether the differences in autophagy regulation in *T. loliiformis* shoots and roots are due to differential energy reserves, we analyzed the sucrose and T6P content in shoots and roots across dehydration and rehydration. Roots contained more sucrose at 80, 60, and 40 RWC compared to shoots (Figure 2A). Roots had 56.26, 65.54, and 53.31% while shoots had 43.74, 34.46, and 46.69% of total plant sucrose at 80, 60, and 40 RWC, respectively (Figure 2B).

**Figure 3:**
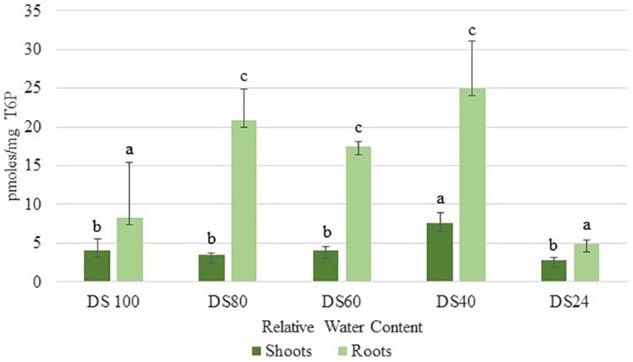
Trehalose-6-phosphate accumulation between shoots and roots of *T. loliifonrtis* during dehydration. *P* < 0.05. Samples denoted with the same letter were not statistically different from each other using a *P*-value < 0.05.

In addition to sucrose, roots accumulated significant levels of T6P during the initial stages of dehydration (80% RWC) (Figure 3). These levels peaked at 40% RWC following a slight drop at 60% RWC (Figure 3). These levels were approximately three- and five-fold more than those detected in shoots at 40 and 60 RWC respectively (Figure 3). These results suggest *T. loliiformis* roots maintain sufficient energy reserves throughout the dehydration process and that these energy reserves may negate the need to activate autophagy pathways in roots.

### Source/Sink Movement of Metabolites Is Aided by *sweet* Genes

Photoassimilates are allocated to plants by transport of sucrose from the photosynthetic to non-photosynthetic tissues for growth, development and response to environmental stress. Sucrose transporters are important in transporting sugars across a membrane (Braun et al., 2014). We assessed whether *T. loliiformis* also utilizes *sweet* genes for transport of sucrose from shoots to roots. Dehydrating and desiccating shoots accumulated transcripts related to *sweet* genes as shown in Table 3. *SWEET 12, 13, 14*, and *15* were upregulated in shoots while *SWEET 15* was upregulated in roots. In combination with the metabolomics data, these results indicate that *T. loliiformis* continues to use the roots as a sink during drought stress and that it uses the *sweet* genes to translocate sucrose from shoots to roots.

**Table 3:** Fold change of sugar transporter *sweet* genes at different desiccation states between shoots and roots of *T. loliiformis.*

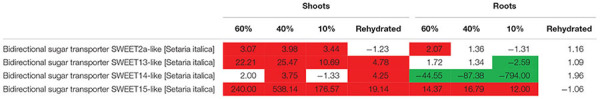

*Cells denoted in red and green indicate increased and decreased transcript accumulation compared to hydrated controls*.

## Discussion

*Tripogon loliiformis* utilizes sugar metabolism and autophagy pathways as one of numerous strategies to facilitate desiccation tolerance in shoot tissues. Upon dehydration, Tripogon shoots and roots are subjected to similar levels of water deficit. In this study we investigated whether *T. loliiformis* roots utilize a similar strategy to shoots, including the activation of autophagy pathways, to survive desiccation. In summary, we show that although roots undergo equivalent rates of water loss to shoots, autophagy is not induced. We postulate that the differential autophagy responses of *T. loliiformis* shoots and roots to dehydration are due to the efficient maintenance of energy reserves and the continued use of roots as sink tissues during dehydration.

Restriction of shoot growth, continuous root growth and modified source-sink metabolite transport are observed in sensitive and tolerant plants in response to water deficit (Lebon et al., 2006). Studies have shown however that the response elicited is dependent on the strength and duration of the stress (Kölling et al., 2015; Durand et al., 2016). *Arabidopsis thaliana* plants subjected to short term water deficit translocate carbon/sugars to young leaves which are more effective at producing energy in water limited conditions (Kölling et al., 2015; Durand et al., 2016). In contrast, when subjected to long-term drought, in addition to translocating carbon to young leaves, *Arabidopsis* promotes increased water uptake by transporting carbon resources to the roots (Durand et al., 2016). Our metabolomics data suggest that *T. loliiformis* elicits a response reminiscent to long-term drought responses in *Arabidopsis* during the early stages of water deficit. Previous studies suggest that resurrection plants accumulate stress-related metabolites in the hydrated state (Urano et al., 2017). This accumulation of stress-associated metabolites helps explain why resurrection plants are typically slow growing, however, it may also prime resurrection plants to respond rapidly to water deficit. We postulate that since *T. loliiformis* is already accumulating stress-associated metabolites when hydrated it is displaying a “pseudo” short-term drought stress response. As such, dehydration triggers the early onset of a “long-term” drought response. The early onset of long-term drought responses by *T. loliiformis* may have at least two physiological consequences. By translocating metabolites away from the shoots, the subsequent low energy stored may cause the early shutdown of metabolism including the shutdown of photosynthesis to prevent water loss and activation of cytoprotective autophagy pathways to obtain nutrients (Williams et al., 2015). Additionally, the transport of carbon resources helps maintain energy balance in the roots, in doing so providing the necessary resources required for increased and continued uptake of water from the soil. This in turn may slow down dehydration and provide the plant with precious time to prepare additional physiological responses required to survive desiccation.

Autophagy is a pro-survival mechanism used by eukaryotes to reinstate homeostasis during sugar and nitrogen starvation as well as other stresses. In plants, autophagy has been linked with delayed leaf senescence and prolonged lifespan (Bassham, 2007; Xiong et al., 2007; Li et al., 2014; Masclaux-Daubresse et al., 2017). Autophagy is characterized by the upregulation of *atg* transcripts, membrane blebbing, the formation of double membrane vesicles known as autophagasomes, partial chromatin condensation and a lack of apoptotic-like DNA laddering (Bassham, 2007). Drought stress interferes with cellular homeostasis through the accumulation of cellular toxins and damaged components (Han and Yang, 2015). Excessive accumulation of these toxins compromises the cells metabolic efficiency and if left unchecked can lead to cell death. Studies have demonstrated cytoprotective roles for autophagy by removal of damaged and misfolded proteins. An additional benefit is the recycling of the nutritional building blocks that comprised the degraded material to the rest of the cell (Maiuri et al., 2007; Thorburn, 2008). Autophagy (atg) transcripts were significantly enriched in *T. loliiformis* shoots but not roots suggesting that shoots initiate autophagy in an attempt to maintain cellular homeostasis during desiccation. Apart from utilizing autophagy to maintain homeostasis, the unfolded protein response (UPR), a signaling pathway activated by the accumulation of unfolded proteins in the endoplasmic reticulum (ER) also acts to relieve stress during drought in plants (Deng et al., 2013; Williams et al., 2014). We therefore postulate that as a mitigative factor, resurrection plants utilize the UPR pathway as a short-term response to effects of drought stress. However, when faced with long-term stress, the UPR pathway is not sufficient and autophagy is activated. Depending on the severity of drought stress, both the UPR and autophagy pathways aim to relieve the damaging effects on the cell. The downregulation of the UPR genes, upregulation of atg genes and abundance of autophagosomes in *T. loliiformis* shoots suggest that activation of autophagy is necessary to withstand desiccation tolerance.

During drought stress, resurrection grasses shut down photosynthesis early to prevent water loss and prepare physiologically for survival (Mundree et al., 2002; Farrant et al., 2007; Challabathula et al., 2016). Sensitive plants however continue to photosynthesize and once stress is perceived, early onset of senescence and flowering are initiated (Kenney et al., 2014; Kazan and Lyons, 2016). In addition to triggering long-term rather than short-term dehydration responses, resurrection plants do not senescence upon dehydration (Griffiths et al., 2014). Unlike *Arabidopsis, Tripogon* can withstand desiccation in its vegetative tissues and only produce seeds once favorable conditions are restored (Williams et al., 2015). We postulate that transport of metabolites to *T. loliiformis* roots rather than channeling resources into reproductive tissue for seed production facilitates desiccation tolerance. Trehalose-6-phosphate (T6P) is a strong indicator of cell energy status regulating carbohydrate metabolism and is associated with drought tolerance (Reape et al., 2008; Kabbage et al., 2017). Dehydrating *T. loliiformis* roots maintained their T6P levels while the shoots did not. The T6P levels are indicative of the roots energy status. High T6P levels in roots indicates the maintenance of high energy status that possibly extends stable metabolism even during stressful conditions. Contrary to roots, *T. loliiformis* shoots had their energy status compromised as shown by the decrease in T6P levels during dehydration. On sensing changes in the energy status, *T. loliiformis* shoots utilize alternative sources of energy thereby switching from anabolism to catabolism. Protein kinases, especially snRK1 protein family regulate energy metabolism in eukaryotes by detecting levels of cellular T6P. Not only does SnRK1 detect energy levels of T6P but also, activation of SnRK1 plays a significant role in energy metabolism in plant stress responses (Baena-gonzalez, 2010). High levels of T6P inhibit snRK1 and catabolism and promote anabolism in emerging plant tissues (Qixian et al., 2009; Sonnewald et al., 2011; Nunes et al., 2013). It is feasible to postulate that the elevated levels of T6P detected in *T. loliiformis* roots inhibit catabolic activity, including autophagy (Lastdrager et al., 2014). In contrast, *T. loliiformis* contained low T6P levels resulting in the activation of snRK1 and autophagy, it also inhibited plant growth thus preparing the shoots for rapid growth recovery, within 72 h, once the environmental conditions become stable (Nunes et al., 2013).

Distribution of carbon within plant organs is important and plays a role in storage, defense and repair (Savage et al., 2016). Most crop plants translocate their carbon from source tissues to sink tissues upon drought stress as a defense mechanism (Yu et al., 2015). Source/sink carbon allocation and cell energy status are key players in plant stress response to drought (Yu et al., 2015). *T. loliiformis* shuts down photosynthesis early upon sensing water deficit and channels the movement of carbon reserves from the shoots to the roots. Translocation of sucrose is an important process that is aided by Sugars Will Eventually be Exported Transporters (*SWEET)* genes. These genes, specifically AtSWEET10, 13, 14, and 15 have been associated with sucrose transport in *Arabidopsis* (Chen, 2014; Patil et al., 2015). Fully hydrated shoots had more carbon levels which diminished during dehydration while hydrated roots had lower carbon levels which increased upon the onset of dehydration. There was however an equilibrium that was maintained between shoots and roots during severe dehydration. Maintaining an equilibrium is critical in gaining desiccation tolerance. Plants that cannot obtain this equilibrium may not survive further dehydration. Although the roots cells were subjected to equivalent levels of water loss, the accumulation of carbon coupled with sucrose homeostasis protected the roots from the effects of desiccation.

**Figure 4:**
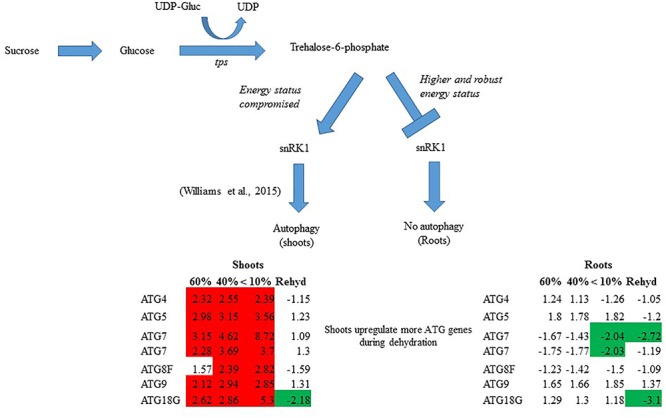
Model for *T. loliiformis* shoot and root response to water deficit based on caloric shift. Roots maintain their energy status during dehydration hence are well-protected. Shoots have their energy compromised and therefore employ mechanisms like autophagy to mitigate adverse effects of drought. Cells denoted in red and green indicate increased and decreased transcript accumulation compared to hydrated controls.

In summary, *T. loliiformis* shoots and roots experience similar levels of dehydration during water deficit but elicit starkly different responses. To minimize water loss and increase water uptake, *T. loliiformis* shuttles energy reserves from shoots to roots during dehydration. Unlike shoots, dehydrating *T. loliiformis* roots maintain their energy status and homeostasis and do not activate autophagy (Figure 4). The continued use of roots as a sink during stress is reminiscent of long-term water deficit responses. Unlike sensitive plants, the accumulation of stress-associated metabolites in resurrection plants, even in hydrated conditions, may prime the plant to an elevated long-term response to mild water loss. An understanding of the mechanisms of sugar sensing and signaling in resilient plants like *T. loliiformis* provides further insight into mechanisms utilized for desiccation tolerance and can aid in developing drought tolerant crops of economic importance. The early elicitation of long-term drought responses in crops may substantially improve their capacity to tolerate stress.

## Author Contributions

PA, BW, LM, UR, and SM conceived and designed the experiments. PA, BW, IN, LM, and TR performed the experiments. PA, BW, IN, LM, TR, UR, and SM analyzed the data. PA, BW, TR, LM, and SM wrote the manuscript.

## Conflict of Interest Statement

The authors declare that the research was conducted in the absence of any commercial or financial relationships that could be construed as a potential conflict of interest.
