# Supplementary material for: Roots of the Resurrection Plant Tripogon loliiformis Survive Desiccation Without the Activation of Autophagy Pathways by Maintaining Energy Reserves
Source: Front Plant Sci. 2019 Apr 25;10:459. doi: 10.3389/fpls.2019.00459 (PMC6494956; doi:10.3389/fpls.2019.00459)
Supplement: Supplementary file 1 [file Data_Sheet_1.PDF]

S1 Table: Validation of autophagy transcripts using qRT-PCR between *T.loliiformis* shoots and roots.

		RWC	ATG18 (Tl_17832)	ATG7 (Tl_19249)	ATG8f (Tl_33291)
Shoots	qRTPCR	60%	6.08	3.50	2.50
		40%	3.49	3.46	1.16
		10%	2.60	4.16	-1.25
		Reh	1.68	1.25	-1.10
	RNA_seq	60%	2.62	2.28	1.57
		40%	2.86	3.69	2.39
		10%	5.3	3.7	2.82
		Reh	-2.18	1.3	-1.59
Roots	qRTPCR	60%	1.43329423	1.8784009	4.635467
		40%	1.41571013	1.5090518	3.81208
		10%	0.388046944	1.0295689	3.413685
		Reh	2.030736565	1.4586034	1.968978
	RNA_seq	60%	1.29	-1.67	-1.23
		40%	1.3	-1.43	-1.42
		10%	1.18	-2.04	-1.5
		Reh	-3.1	-2.72	-1.09

S2 Table: List of q-PCR primers and their target sequences

Target	Primer name	Sequence 5'-3'
ATG18	TL_17832F	CACCAGCAACAACCAGAAGC
	TL_17832R	TCAAAACGTGATGGCCCAGT
ATG7	TL_19249F	CCAACTCTTCTCTGCGCTGA
	TL_19249R	ACAGCTTATCAGTGGCAGCA
ATG8F	TL_33291R	ATCCCGATCGCATTCCTGTC
	TL_85130R	GCGCGTTGATGATGGAGAAC
